# Perceived causes of mental illness and views on appropriate care pathways among Indonesians

**DOI:** 10.1186/s13033-021-00497-5

**Published:** 2021-09-23

**Authors:** Sabrina Gabrielle Anjara, Carol Brayne, Tine Van Bortel

**Affiliations:** 1 Cambridge Public Health, University of Cambridge School of Clinical Medicine, Cambridge Biomedical Campus, Forvie Site, Robinson Way, Box 113, Cambridge, CB2 0SR UK; 2 Faculty of Health and Life Sciences, De Montfort University, Leicester, LE2 7GZ UK

**Keywords:** Colonial society, Colonial mental health care, Low and middle-income countries, Public perception, Mental health perceptions, Qualitative study, Indonesia

## Abstract

**Background:**

The mental health system in Indonesia comprises attempts to modernise a colonial relic. There is still a disconnect between available services and help-seeking behaviours at the grassroots level. This study aims to explore the perceptions of Javanese people on the aetiology of mental illness and their ideas on how to deal with individuals who may have mental illness.

**Methods:**

This qualitative study involves semi-structured interviews, embedded in a cluster randomised trial examining the clinical and cost-effectiveness of primary mental health services. Interviews were conducted with Indonesian and Javanese. The recruitment procedure was aligned to the trial. Participants were primary care patients recruited from 21 sites across Yogyakarta province. Interviews were recorded, transcribed, and translated into English. Thematic analysis was used to analyse the interview transcripts.

**Results:**

75 participants took part in the study: 51 women (68%) and 24 men (32%). Key themes emerged around perceived causes of mental health problems (including ‘extrinsic factors’; ‘intrinsic factors’; and ‘spiritual factors’), and perceived appropriate pathways of care (‘modern medical science’; ‘social support and activities’; and ‘religious or spiritual interventions’). Gender potentially influenced some of the responses.

**Conclusions:**

Themes indicate the variety of preconceptions towards mental health problems and assumptions regarding the best management pathways. Some of these preconceptions and assumptions support the utility of modern medical care, while the rest promote spiritual or religious healers. Participants’ ideas of the appropriate care pathways largely correspond to their perception of what the symptoms are caused by. Despite hints to some understanding of the bio-psycho-social model of mental illness, most participants did not capture the complexity of mental health and illness, indicating the importance of contextual (especially culturally and religiously-aligned) public education around mental health, illness and care.

**Supplementary Information:**

The online version contains supplementary material available at 10.1186/s13033-021-00497-5.

## Background

Indonesia is a South-East Asian archipelago of over 17,000 islands inhabited by around 270 million people who belong to more than 300 ethnic groups and speak more than 500 languages. Psychiatry was introduced to Indonesia during the Dutch colonial period [1]. Over half of Indonesia’s population live on the island of Java, while those with Javanese ethnicity amount to around 40% of the total population. In 1868, Bauer and Smit surveyed mental health care in the Netherlands East Indies and concluded that in comparison to the situation in Europe, colonial psychiatric care was inhumane and unscientific, lacking proper psychiatric facilities [2]. As a result, many people with mental health problems did not receive any medical treatment and were kept in chains in their villages. Bauer and Smit persuaded the Dutch government to restructure the ‘treatment of the insane’ in the Netherlands East Indies according to modern European principles, leading to the establishment of several transit houses, agricultural colonies and a stand-alone mental asylum in Java [3].

Between 1882 and 1938 mental health care in the archipelago developed alongside European psychiatry and expanded in bed capacity [4]. To successfully treat native patients, European psychiatrists often emphasised the need for study of the native mind [5, 6], the development of diagnostic tools and the adaptation of therapeutic methods to the needs of “native patients”, whose needs often differed from their European counterparts [7]. Emil Kraepelin’s research in Java resulted in several interesting observations. He discovered that tertiary syphilis and alcohol-related mental disease were absent among the Javanese. He also noticed that Javanese patients had a shorter duration of depressive episodes, but prolonged and highly expressive manic episodes and that they have a significantly better prognosis compared to European patients.

Following Dutch physicians’ reports that the Javanese were highly instinctive, suggestible, emotionally charged, while displaying laziness and poor development of secondary functions and individuality [8]. Indonesian physicians protested and argued that the clinical investigation of Indonesian individuals with mental health problems should be conducted by physicians who speak their language and were aware of their cultural conventions [3]. More recently, a comparison of schizophrenia symptoms between Indonesian and British patients found that Indonesian patients suffer more from over-activity and less from delusions, hallucinations, and depression [9]. Despite the awareness of transcultural differences in the presentation of symptoms, Western concepts of care (i.e., institutionalisation) were imposed in the belief that they were appropriate for the task of colonial nation-wide health services provision [10].

Post-independence, the Indonesian psychiatric curriculum was aligned with the United States model [1]. Henceforth, Indonesian mental health care was primarily influenced by American psychiatry, laying the foundation for new, open-style hospitals, and outpatient care. The bio-psycho-social model became the basis of psychiatric thinking since the mid-1960s [11]. Regardless, the medicalisation of mental health problems tends to disregard the subjective experience of the illness in favour of providing accessible mental health treatment within a complex health system (in Indonesia, this is managed by the Directorate of Mental Health). With very few psychiatrists in the country, people in remote areas have limited access to treatment.

The disconnect between the services offered and the services needed and accepted has been recognised for decades [10]. A study looking at traditional healing methods found that up to 80% of people with psychiatric conditions would visit traditional healers before consulting physicians [12]. This study led to a national policy acknowledging the co-existence of traditional healers and medical doctors in providing care to the mentally ill. Regardless, the mental health treatment gap in Indonesia remains high (e.g., in 2005 the treatment gap for psychotic disorder in West Java was 96.5% [13]) and many people with untreated mental health problems were still shackled or caged by their family members.

*Pasung* is the Indonesian term for the practice of shackling or caging persons perceived to have mental health problems. Despite *pasung* having been highlighted in Bauer and Smit’s 1868 report to the colonial government and outlawed in Indonesia since 1977, the practice continues today. The government estimated that over 57,000 individuals, mostly in rural areas, have suffered this inhumane treatment, often at the hands of family members and religious or traditional healers [14]. Despite the ‘Free from *Pasung*’ programme set up in 2010, the Human Rights Watch estimated that 18,000 Indonesians were in *pasung* at the time. Human rights violations towards the mentally ill, even in the modern era, have been considered “a failure of humanity” [15].

At the time of the writing of this manuscript, there were several care options for mental health issues: colonial-era psychiatric institutions exist alongside American-style outpatient psychiatry clinics embedded in general hospitals. There are provinces without psychiatric institutions and even psychiatrists. The country’s network of almost 10,000 primary care centres or community health centres (*Puskesmas*) stepped up to shoulder this burden. In 2015, 30% of *Puskesmas* provide mental health care at the community level, providing public education, counselling, basic psychiatric services, house visits, community outreach, and referral to specialist care [16]. These were carried out by nurses who had undergone certification in Community Mental Health Nursing, or general practitioners stationed in a *Puskesmas*. More recently, some districts in Indonesia have started employing clinical psychologists to be based in *Puskesmas*.

The expansion of Western-style mental health services continues, with more districts in Indonesia adopting the framework that places clinical psychologists in primary care centres. In 2015, the Indonesian Ministry of Health began training primary care doctors in the WHO Mental Health Gap Action Programme (WHO mhGAP), a curriculum on mental health services in primary care [17]. This allowed people with mental health concerns to access mental health care at their nearest Puskesmas, at very low costs or free of charge, depending on levels of subsidy set by district governments [16]. While the programme is being implemented in many low- and middle-income countries there remains limited information on whether the target audiences are aware of the availability of these services, aligned to the philosophy of these services, or find it socially acceptable to access these services.

The enormous treatment gap highlights the disconnect between available services and help-seeking behaviour at the grassroots level. While quality, cost, and convenience of modern medicine are often the drivers of implementation, cultural compatibility should be seriously considered [18]. Culture significantly influences health-related beliefs, behaviours, and values [19]. In the Javanese experience of mental illness, religious beliefs, spiritual ideas, and modern medicine intertwine [20]. Although Indonesia has the largest Muslim population in the world, many people in rural areas practice a hybrid of Islam and traditional animism [21]. This pervasive belief in superstitions and animism, inherent in local traditions, has been seen as an obstacle to spreading medical insight at the grassroots level [3].

While Java is 90% Muslim, many were born into *Abangan* families, who identified as Muslim but held on to folk beliefs and cultural practices prevalent in the region before the arrival of Islam [22]. As a result of their spiritualism, people with mental health problems are usually taken to a traditional healer when their symptoms become intolerable for family members [10]. Decades have passed and the communal mindset may have changed. With the shift towards a puritan form of Islam (Santri Islam instead of Abangan, [22]) where visits to shamans and nature worship are considered blasphemous, modern medicine has become increasingly acceptable in Indonesian communities alongside the decline in the number of traditional healers around. Yet, there has been no study that explores the subjective understanding of mental health problems among those who have used modern medical facilities.

This study aims to explore the subjective understanding of the causes of mental health problems and thoughts around appropriate care among Javanese people who reached out to modern medicine for their physical ailments. In recognition of the role of culture in shaping perceptions, the importance of the Javanese patient’s point of view will be explored within the context of their religion, moral values, and everyday practices [23]. Linking ‘disorders’ with ‘subjectivity’ has the potential for increasing the understanding of the lived experience of persons in complex, threatening, and uncertain conditions [24] and as such their preferred pathways of care within the context of their cultural, religious, and modern understanding of the conditions. Understanding the ‘subjectivity’ improves the effort to provide accessible and accessed mental health services.

## Methods

### Design

The study was embedded into a partially randomized cluster trial which examined the clinical and cost-effectiveness of two frameworks of primary mental health care in Yogyakarta, Indonesia: the WHO mhGAP framework and the Specialist Co-location model [17]. In the WHO mhGAP framework, primary care doctors received add-on training in mental health management in primary care and are expected to diagnose and manage several mental health problems within primary care. In the Specialist Co-location model, clinical psychologists are employed in *Puskesmas* and receive walk-in patients as well as referral from primary care doctors.

As this study was embedded into an existing trial, the recruitment period for the current study was designed to coincide with the recruitment period for the trial, and the study locations were identical. Interviews conducted were semi-structured with a succinct topic guide (Additional file 1) translated to Bahasa Indonesia. The interviews were designed to be around 15–30 min in duration.

The interviews were conducted in December 2016 by three trained research assistants (an anthropologist, a clinical nurse, and a psychologist) at 21 sites across all five districts in Yogyakarta, one of 34 provinces in Indonesia. The sites were chosen following stratified randomisation based on the urban/rural divide and population size of the districts [17]. Pre-recruitment meetings between the interviewers and the lead author (SGA) allowed them to run through the Topic Guide (Additional file 1) and role-play the interviews. All interviewers had daily debriefings with SGA—a native speaker of the language and chartered psychologist—who was on-site during the study period and present at several random interviews to ensure the consistency of interview styles between interviewers. All interviewers met in person after the first week of interviews (about six interviews) to discuss challenges and approaches to conducting the interview in Javanese language with older participants. About halfway through the recruitment, all interviewers met in person again to identify gaps in demographics. The meetings did not alter the topic guide.

### Procedure

All adult primary care patients (above 18 years of age) seeking care for their physical ailments at a *Puskesmas* were eligible to participate. Family members of patients were excluded from participation. Participants would have completed the 12-item General Health Questionnaire as part of a trial to which this present study was embedded [25]. Interview participants were approached at a primary care centre—who acted as gatekeepers—where a research assistant explained the purpose of the study, provided an information sheet, and invited potential participants to ask questions before deciding whether to participate. Upon receiving signed consent, the research assistant would arrange for the interview to take place at a suitable time and place. Participants were informed that their input was entirely confidential, that the results of the study would be de-identified, and that they could terminate their participation at any point. They were also informed that declining to participate would not affect their continuation of care. Interviews were audio-recorded and transcribed verbatim.

### Analysis

The interviews were translated into English by a licensed translator, preserving the sentence structures and irregular words. The translated transcripts were re-checked and then analysed by a bilingual researcher (SGA) to ensure the reliability of the translation. SGA coded all 75 transcripts in English using NVivo 12. SGA also coded a third of the transcripts (N = 25) in the original language to ensure the alignment of the analysis of the translated transcripts to the meaning and nuances of the original version. The transcripts were analysed using thematic analysis, in line with Braun and Clarke’s (2006) guidelines. The analysis was guided by the framework of the topic guide, in which participants were asked about their background, their perceptions of the causes of mental health problems, and their thoughts on how best to manage symptoms of mental health problems (see Additional file 1). Codes and themes that emerged from the data were further developed through a collaborative and iterative process with other co-authors, during which confluence was achieved. This discursive co-production approach supported the quality and rigour of the analysis and trustworthiness of the results, by comparing codes and themes and reaching a consensus on the results of the analysis.

Quotes extracted from the transcripts might require re-translation if there were extensive use of irregular words, colloquial language, or abbreviations. In such cases, quotes might also be adjusted to align with the English grammar (past tense). Any additions to the quotes are based on the context of the full interview and are indicated using square brackets [].

All authors were immersed in the data and involved in the analysis throughout the project to increase rigour. Throughout, all authors engaged in a process of active reflexivity (reviewing fieldnotes; assessing and documenting the research assistants’ perspectives in the research; developing research assistants’ techniques and methods to further explore discoveries; meeting regularly to discuss the results) to examine the authors’ roles and influence on the interpretative process, which is recognised as imperative in improving the quality and rigour of qualitative research. The importance of active reflexivity throughout the analysis is underpinned by a constructivist philosophy, whereby any knowledge or findings identified through the data collection and analysis are informed by the active involvement of not only the participant but also the researchers in generating the data, which is informed by the lived experience of both.

## Results

In total, 75 adult primary care patients participated in the study: 51 women (68%) and 24 men (32%). Their age ranged between 18 and 78 (Table 1) with the median age range of 30–45. Most respondents (N = 66, 88%) were Muslims. According to the census, 90.96% of Yogyakarta’s residents are Muslims. In 2016, the province was home to 3.7 million residents, the majority of whom (31.7%) lived in the Sleman regency. Of all our participants, 40 (53.3%) lived in the Sleman regency.

**Table 1:** Participant characteristics (n = 75)

Characteristics	Total frequency	Female (n = 51, 68%)	Male (n = 24, 32%)
Age			
18–29	26 (34.7%)	17 (33.3%)	9 (37.5%)
30–44	18 (24.0%)	12 (23.5%)	6 (25.0%)
45–59	23 (30.7%)	17 (33.3%)	6 (25.0%)
60 and above	8 (10.7%)	5 (9.8%)	3 (12.5%)
Education			
Primary school	13 (17.3%)	11 (23.4%)	2 (9.1%)
Junior high school	14 (18.7%)	12 (25.5%)	2 (9.1%)
High school certificate	27 (36.0%)	19 (40.4%)	8 (36.4%)
Diploma	5 (6.7%)	1 (2.1%)	4 (18.2%)
Undergraduate degree	9 (12.0%)	3 (6.4%)	6 (27.3%)
Masters degree	1 (1.3%)	1(2.1%)	0
Unknown	6 (8.0%)		
Religion			
Islam	66 (88.0%)	50 (98%)	16 (66.7%)
Christian/roman catholic	9 (12.0%)	1 (2.0%)	8 (33.3%)
District of Domicile			
Bantul	13 (17.3%)	11 (21.6%)	2 (8.3%)
Gunungkidul	1 (1.3%)	1 (2.0%)	0
Kota	14 (18.7%)	6 (11.6%)	8 (33.3%)
Kulon Progo	7 (9.3%)	5 (9.8%)	2 (8.3%)
Sleman	40 (53.3%)	28 (54.9%)	12 (50.0%)

A large proportion of participants were educated below high school level (36%) or had attained a high school certificate (36%). Only 20% had professional diplomas or university degrees. Six participants declined to comment on their educational background. Male participants tended to be better educated than their female counterparts, χ^2^(5) = 14.5, p < 0.05. Age was not found to be significantly related to education (Table 2).

**Table 2:** Participants’ education, frequency by age groups (n = 69)

Age	Primary	Junior high	High school	Diploma	University	Masters
18–29 years (n = 24)	1	2	12	3	5	1
30–44 years (n = 16)	3	5	6	0	2	0
45–60 years (n = 22)	6	7	6	2	1	0
Above 60 years (n = 7)	3	0	3	0	1	0

The interviews ranged between 15 and 80 min in duration (Mean of 23.3 min, SD = 10.9; Median of 19 min). While interviews were designed to take between 15 and 30 min, 20% of interviews were over 30 min in duration. In these longer interviews, participants took longer to develop rapport with the interviewer. Despite the discrepancy in interview duration, the shorter interviews addressed all the points in the topic guide. Only one interview was above 45 min, as the participant provided extensive unsolicited anecdotes about their children and grandchildren.

During the trial in which this study was embedded, primary care patients were invited to see a clinical psychologist if they indicated any symptoms of mental health problems in the 12-item General Health Questionnaire which was being validated for use in primary care [25]. Forty of 75 participants interviewed had indicated at least one symptom of mental health problems in the screening questionnaire and had seen a primary care psychologist before the interview. Only eight of the forty received a formal diagnosis (six females, two males; all Muslims). The interviews explored participants’ perceived causes of mental health problems in general, rather than what they were diagnosed with.

The codes and themes emerging from the data were examined across participant demographics, leading to the inference that all themes were described across all five districts within the province, and across gender. The subthemes were not associated with district or religion. Some of the subthemes might be related to gender, as indicated in Tables 3 and 4. Age was found to be correlated to responses supporting traditional healing practices, with older participants more likely to have accessed and experienced traditional healers. Themes within the two core areas (**perceived causes** and **perceived best care pathway**) are presented in *italics*. Subthemes are presented with single quotation marks, and participant quotes are indicated using double-quotes.

**Table 3:** Themes on the perceived causes of mental health problems and their frequency of mention

Extrinsic factors					
‘Intensity of pressure from family or problems’ (n = 21)					
Female	Male	18–29	30–44	45–60	above 60
14	7	7	6	7	1
66.70%	33.30%	33.30%	28.60%	33.30%	4.60%

‘Inability to cope with stress’ (n = 17)					
Female	Male	18–29	30–44	45–60	above 60
12	5	7	4	5	1
70.60%	29.40%	41.20%	23.50%	29.40%	5.90%

‘Quantity of problems’ (n = 4)					
Female	Male	18–29	30–44	45–60	above 60
3	1	1	1	1	1
75%	25%	25%	25%	25%	25%

‘Economic or environmental factor’ (n = 8)					
Female	Male	18–29	30–44	45–60	above 60
4	4	3	1	2	2
50%	50%	37.50%	12.50%	25%	25%

‘Lack of social support’ (n = 4)					
Female	Male	18–29	30–44	45–60	above 60
4	0	3	1	0	0
100%		75%	25%		

Intrinsic factors					
‘Thinking too much’ (n = 22)					
Female	Male	18–29	30–44	45–60	above 60
13	9	6	7	7	2
59.10%	40.90%	27.30%	31.80%	31.80%	9.10%

‘Congenital defects or hereditary factor’ (n = 3)					
Female	Male	18–29	30–44	45–60	above 60
1	2	2	0	0	1
33.30%	66.70%	66.70%			33.30%

‘Inability to control emotions’ (n = 2)					
Female	Male	18–29	30–44	45–60	above 60
1	1	1	1	0	0
50%	50%	50%	50%		

‘Issue of the mind’ (n = 5)					
Female	Male	18–29	30–44	45–60	above 60
4	1	2	2	1	0
80%	20%	40%	40%	20%	

‘Lack of mental strength’ (n = 3)					
Female	Male	18–29	30–44	45–60	above 60
2	1	0	2	1	0
66.70%	33.30%		66.70%	33.30%	

‘Resentment from unmet needs or desires’ (n = 7)					
Female	Male	18–29	30–44	45–60	above 60
4	3	3	1	3	0
57.14%	42.86%	42.86%	14.28%	42.86%	

‘Negative thoughts’ (n = 2)					
Female	Male	18–29	30–44	45–60	above 60
2	0	1	1	0	0
100%		50%	50%		

‘Narrowmindedness’ (n = 1)					
Female	Male	18–29	30–44	45–60	above 60
1	0	0	0	1	0
100%				100%	

Spiritual factors					
‘Daydreaming and satan’ (n = 5)					
Female	Male	18–29	30–44	45–60	above 60
3	2	1	2	1	1
60%	40%	20%	40%	20%	20%

‘Lack of interaction with god’ (n = 2)					
Female	Male	18–29	30–44	45–60	above 60
2	0	1	1	0	0
100%		50%	50%		

‘Santet (black magic)’ (n = 2)					
Female	Male	18–29	30–44	45–60	above 60
1	1	2	0	0	0
50%	50%	100%

**Table 4:** Themes on the perceived appropriate treatment pathways and frequency of mention

Modern medical and health science					
‘Institutionalisation in a psychiatric hospital’ (n = 18)					
Female	Male	18–29	30–44	45–60	Above 60
11	7	9	5	2	2
61.10%	38.90%	50.00%	27.80%	11.10%	11.10%

‘Seeking help from a psychiatrist’ (n = 7)					
Female	Male	18–29	30–44	45–60	Above 60
5	2	1	3	2	1
71.40%	28.60%	14.30%	42.90%	28.60%	14.30%

‘Seeking help from a psychologist’ (n = 12)					
Female	Male	18–29	30–44	45–60	Above 60
8	4	6	1	3	2
66.70%	33.30%	50.00%	8.30%	25.00%	16.70%

‘Seeking help from a primary care doctor’ (n = 14)					
Female	Male	18–29	30–44	45–60	Above 60
11	3	4	1	9	0
78.60%	21.40%	28.60%	7.10%	64.30%	

Activities or social support					
‘Family support’ (n = 7)					
Female	Male	18–29	30–44	45–60	Above 60
6	1	3	2	2	0
85.70%	14.30%	42.90%	28.60%	28.60%	

‘Social support outside the family’ (n = 9)					
Female	Male	18–29	30–44	45–60	Above 60
5	4	1	3	4	1
55.60%	44.40%	11.10%	33.30%	44.40%	11.10%

‘Relaxing activity’ (n = 5)					
Female	Male	18–29	30–44	45–60	Above 60
3	2	2	1	0	2
60%	40%	40%	20%		40%

Religious or spiritual intervention					
‘To pray more’ (n = 10)					
Female	Male	18–29	30–44	45–60	Above 60
7	3	1	3	4	2
70%	30%	10%	30%	40%	20%

‘Seeking help from an Islamic teacher’ (n = 8)					
Female	Male	18–29	30–44	45–60	Above 60
4	4	3	2	3	0
50%	50%	38%	25%	38%	

‘Seeking help from a shaman’ (n = 5)					
Female	Male	18–29	30–44	45–60	Above 60
4	1	1	2	2	0
80%	20%	20%	40%	40%	

Others					
‘*Pasung*’ (n = 1)					
Female	Male	18–29	30–44	45–60	Above 60
0	1	0	0	1	0
	100%			100%	

### Perceived causes of mental health problems

Three main themes were identified describing participants’ perception of the causes of mental health problems: *extrinsic factors*, *intrinsic factors*, and *spiritual factors* (see Table 3).

The theme ***Extrinsic factors*** refers to potential causes of mental health problems in the form of external or environmental influences, including the challenges of daily life, financial difficulties, illnesses beyond one’s control, and/or a combination of several external influences. Participants consistently reported *extrinsic factors* which includes the inability to cope with ‘stress’ or the quantity and intensity of problems, as potentially causing mental health problems. Individuals could face issues from several aspects of life (‘quantity of problems’) which made managing them more complex. Participants generally perceived the importance of having friends and family to discuss one’s issues with. A ‘lack of social support’ therefore could precipitate mental health problems, as individuals are left to deal with their issues alone.

“Mental health problems may be caused by various things: perhaps they experienced a lot of pressures, they have issues, and maybe they can’t tell anybody, so they keep it to themselves.”

A Female Participant (18–29y).

Economic and environmental factors include the financial pressures of debt, growing up in poverty, and traumatic events. These are often made more complex in a collectivist culture where relatives live within proximity and social comparisons regarding ownership of possessions and income inevitably occur.

“I realised that [for my neighbours], mental health problems were because of their financial condition. There was a [financially] poor sibling who was compared with his older and younger siblings who were more successful. They told me about the comparisons.”

A Male Participant (45–59y).

The theme ***Intrinsic factors*** refers to potential causes of mental health problems resulting from individual shortcomings and deficiencies. Ruminating over life’s challenges, or ‘thinking too much’ (literal translation of *banyak pikiran*) is perceived by many participants to be among the primary causes of mental health problems. *Banyak pikiran* is the common Indonesian phrase used when describing the experience of being overwhelmed by too many problems—similar to the English term cognitive overload. *Banyak pikiran*, especially where the complexity of issues is thought to be beyond one’s mental capacity, has the highest frequency of mention among all perceived causes of mental health problems (22 of 75 participants).

“Maybe they’re thinking about an issue beyond their mental capacity. They could become mentally disturbed if the complexity of their rumination is beyond what they are capable of processing, for instance, their capacity is in here but they think about this instead (demonstrating with his hands). Now, there could be a disorder right?”

A Male Participant (above 60y).

Even when individuals face the ordinary demands of life, participants perceived that other *intrinsic factors* such as having a ‘genetic’ predisposition, having ‘unmet needs or desires’ and the ‘lack of mental strength’ could predispose a person to mental health problems. Resentment from unmet needs or desires arises whenever a person experiences the provisions of goods, services, or opportunities that fall short of their expectations or requirements. The resentment could result in the inability to view problems in light of other aspects of life, which a participant termed “narrowmindedness”, or the ‘inability to control emotions.’ A sizeable number of participants (N = 7) thought an ‘issue of the brain’ could result in mental health problems indicating some understanding of the potential biological aetiology of the symptoms. “Maybe their brain is not strong enough to process their issues” *(A Female Participant (30–44y)).*

The theme ***Spiritual factors*** refers to perceived causes of mental health problems as taught in pre-Islamic traditions and folk beliefs as well as Islamic teachings of good versus evil. Several participants acknowledged that in the Javanese culture, *spiritual factors* are still believed to be the cause of mental health problems. This belief is also common among Muslims in other countries, who attributed psychiatric symptoms to jinn [26]. ‘Daydreaming’ or as a participant said “having an empty mind” could allow passing evil spirits (jinn) or Satan himself to “occupy the head”. Also, the “lack of interaction with God” leaves a person vulnerable to spirits possession. In line with the Javanese folk beliefs rooted in animism, several participants believed that evil spirits could be sent to occupy someone’s body by envious others through Javanese black magic or voodoo called ‘*santet*.’

“The foundation of their faith in God is perhaps not up to standard. If they never do all the requirements expected in the Islamic faith, they are more vulnerable to mental health problems.”

A Male Participant (30–44y).

Several participants were aware of the various schools of thought regarding the aetiology of mental health problems attributed to different sources of information.

“There are various causes. According to the doctor (I just saw), it usually came from the mind. In Javanese belief, it is caused by demon possession. You see, they are different. According to science, it will also be different.”

A Female Participant (18–29y).

One participant’s response highlighted the shifting understanding of the aetiology of mental health problems.

“Nowadays (mental health problems) are thought to be triggered by a lot of thoughts and pressures. In the old times, they are thought to have been caused by spirits. Yes, because of the lack of knowledge about mental health problems in the past.”

A Female Participant (45–60y).

Even though 40 participants had at least one positive response to the 12-items of the screening questionnaire and saw a clinical psychologist for an in-depth psychological assessment before the interviews, only two saw themselves as having mental health problems. These participants (female) had received a formal diagnosis and were able to point to specific incidents which may have precipitated the symptoms, without being asked. The rest did not refer to their symptoms when asked about the causes of mental health problems and were not prompted in any way to refer to their symptoms.

### Perceived appropriate treatment pathways

Participants were asked how to best care for people with mental health problems. Three clear themes emerged from the interview data: *modern medical and health science*, *social support or activities*, and *religious or spiritual intervention* (see Table 4). One participant believed that she was not in a position to decide on the management of mental health problems and believed that the appropriate action is to ‘let the family decide’. Of note, one participant stated that ‘*pasung*’ (shackling or caging someone with mental health problems) was the appropriate course of action.

The majority of participants indicated ***modern medical and health science*** was an appropriate treatment pathway. A third of participants believed that taking people with mental health problems to ‘a psychologist or a psychiatrist’ would be the most way to manage their symptoms. A quarter of all participants believed that ‘institutionalisation in a psychiatric hospital’ would be required as such places are better placed to deal with mental illness. Fourteen participants believed that a ‘seeking help from a primary care doctor’ would be appropriate.

“For individuals with mental illness, a mental hospital can give them an explanation, enlightenment. If they have lost their mind, they can stay over and be [treated] in the hospital.”

A Male Participant (30–44y).

Other participants believed that ***social support or activities*** can alleviate mental health problems. These participants believe that professional help is not required for the more common problems. ‘Friends’ and ‘family support’ could help individuals talk through their issues and manage them. “The closest people should support them, give them love and attention” (*A Female Participant (45–59y)).* Attempting ‘relaxing activities’ such as resting, or a nature walk was perceived to help take one’s mind away from one’s issues.

A fifth of all participants believed in ***religious or spiritual intervention*** to overcome mental health problems, including “praying and seeking forgiveness from God” (*Female Participant (30–44y*)), ‘seeking help from an Islamic Teacher’ or ‘seeking help from a shaman’. An Islamic Teacher or religious leader (*ustad*) could perform Islamic exorcism (*ruqyah*) which is believed to rid individuals of the jinn occupying an individual’s body. A shaman, on the other hand, perform rituals based on traditional Javanese folk beliefs rooted in animism and usually require sacrifices of material possessions or small animals.

“Well, usually individuals with mental health problems go to a shaman. It has been said that going to an adept shaman could heal mental illness…”

A Female Participant (30–44y).

A total of 14 participants had previously visited shamans. Of these, eight participants “recognised the existence of shamans” but were unlikely to go again as they felt it was “against their religious belief,” or feared that “it might backfire”. In their opinion, visiting shamans was forbidden by Islam. “Yes, I used to go to a shaman regularly, but as the time passed by, someone told me it is not allowed. Since then, I stopped” *(A Female Participant (Above 60y)).*

Even younger people in Java still visit shamans although the practice might be frowned upon by those who were more religious. “*So,* my friend once invited me to *nyabeh* (send voodoo to) a person. She said, “Let’s see a shaman.” But I don’t want to be a *shirk* (practicing idolatry) which is forbidden in Islam” *(A Female Participant (18–29y)).*

Six participants—all older—have had experiences with shamans but would no longer go as they felt the shamans were only after money, without providing results.

“In the past, I did go to shamans, Miss. Yes, I believed in them because the purpose of going is to bring all our problem to someone who could help. However, if I have to travel far, sacrifice my time for work and family, leave everything I have to them (money and anything else they asked for), but in the end, nothing worked, then from that point, I surrendered to the one who owns this life (God).”

A Male Participant (45–59y).

On the other hand, two participants, who mentioned that they prayed five times a day, frequent a mosque to study Surah Ya-Sin, and read the Quran regularly at home, also believed that shamans could be Allah’s medium for healing. “Yes, I believe that shamans might have paranormal abilities (*orang pintar*), and they might be a medium from Allah to heal” *(A Female Participant (45–59y)).*

Two participants reported that there are ‘traditional healers’ who are not shamans, but someone more experienced who could direct the patient towards healing. They do not use black magic or require sacrifices but instead provide coaching and advice to those who see them. “It’s like going to an elder person, someone that can give positive advice, and not a shaman” *(A Female Participant (30–44y)).*

Two participants believed that an ‘Islamic teacher’ could provide relief. While only one participant mentioned explicitly that *ruqyah* or Islamic exorcism is the best method to get rid of mental health problems by expelling possessing spirits from the body, two other Muslim participants believe that the Islamic approach could be synergistic with animistic healing practices in treating mental health problems. Six participants, all Muslims, believe that ‘prayer’ is most effective in dealing with mental health problems.

“Don’t do ridiculous things—just pray. If it was me, I would just pray. If we pray, we ask for forgiveness from Allah, do much taubah (seeking forgiveness) that’s it. Inshallah (if Allah wills it), Allah will grant anything we wish.”

A Female Participant (Above 60y).

## Discussion

This study sets out to explore the subjective understanding of the causes of mental health problems and thoughts around appropriate care among Javanese people who reached out to modern medicine. This was in the context of a national scale-up of primary care mental health services and an enormous mental health treatment gap in Indonesia. Information gathered provides a deeper insight into people’s perceptions of mental health, illness and care, and whether or not they will access the mental health services that are being made more accessible by the government and how to overcome potential barriers.

We identified three themes that describe Javanese people’s perceptions of the causes of mental health problems: *extrinsic factors*, *intrinsic factors*, and *spiritual factors*. Extrinsic factors refer to pressures seen as beyond the control of the individual. Intrinsic factors refer to the inability of the individual’s mind to cope with the daily demands of life, leading to mental health problems. These factors may cause rumination or stress, which has been linked to physical and psychological consequences. Among Indonesian academic publications, ‘thinking too much’ has been associated with hypertension [27], poor sleep quality [28] and confusion in the Balinese Hindu tradition [29]. Lastly, spiritual factors refer to evil spirits or jinn occupying the individual’s psyche where space was created while daydreaming or due to religious deficiencies.

Although 40 of 75 participants reported symptoms of mental health problems through a screening questionnaire, findings indicate that very few participants attributed symptoms to human biology, but many more participants had insights on the possible psychological and social factors. This is in line with participants’ own experiences with and observations of affective symptoms which seem to be triggered by psychosocial issues. Few respondents refer to more than one perceived aetiology of mental health problems, indicating most respondents’ limited understanding of the complexity of mental health and illness. Conversely, we expect acknowledgement of multiple causes of mental health problems to reflect a better understanding of the aetiology.

A large proportion of our sample (18 of 75) indicated their preference for those with mental health problems to be remanded in a psychiatric facility—clearly a result of the popularity of colonial-era asylums. Stories passed down the generations might point to asylums as the treatment pathway for mental health problems, primarily because the more modern specialist mental health care could be expensive, and primary mental health care remains largely unknown. Looking more carefully at the age distributions for each subtheme, significantly more participants in the 45–60 years age group indicated their preference to seek help in primary care clinics. They were also more likely to indicate that prayer might help with mental health problems compared to participants in other age groups.

Considering the gender distribution, male participants were more attuned to the potential of economic or environmental factors, or biological factors, in precipitating mental health problems. Female participants were more amenable to seeing their primary care doctors, compared to male participants who were slightly more approving of psychiatric institutions. Female participants were also more likely to seek help within the family compared to male participants who preferred seeking solace outside the family. In the domain of religious interventions, female participants favoured the more solitary approach of prayer compared to male participants who preferred guidance from a religious teacher. These findings indicate vastly different help-seeking approaches between gender.

The data also show that many participants still hold traditional beliefs regarding mental health problems, yet they are increasingly aware of religious restrictions towards some form of traditional healing practices. This is in line with a recent study on the cultural understanding of psychotic illness among caregivers of persons with first-episode psychosis [30]. The awareness of religious aversions towards shamans despite prevalent local beliefs in demon possession and black magic may present a cognitive dissonance which may result in the delay of care-seeking. Both *intrinsic factors* and *spiritual factors,* which accentuate personal weakness and religious deficiencies, may exacerbate the stigma of mental health problems in the community also influencing care-seeking behaviours. Our findings indicate that there is a need for evidence-based and culturally appropriate psychoeducation at the grassroots level.

Despite the varying perception of the causes of mental health problems, half of our participants believe in *modern medical and health science* as the appropriate management pathway. In contrast with older literature which indicates that people with mental health problems are usually taken to traditional healers [10], our findings show that only a small percentage of respondents had gone to see a traditional healer before seeking modern medical treatment. This is not unexpected, as access to information has improved over the last few decades, in conjunction with campaigns to reduce the stigma of mental illness by providing public health education. Some participants believe that *relaxing activities* or *social support* may help alleviate mental health problems. The belief in *religious or spiritual intervention* remains pervasive with a fifth of respondents indicating these to be an appropriate remedy for mental health problems. The belief in religious or spiritual intervention was salient among our participants, across religion, gender and age groups, contrary to our a priori expectations. Seeking help from religious leaders for mental health problems is a phenomenon not limited to Indonesia, but is also prevalent among Muslims in Europe [26], as well as Rohingya refugees [31]. Worryingly, one participant considered ‘pasung’ (shackling of people with mental health problems) to be an appropriate way of managing mental health problems, despite a concerted effort to eliminate *pasung* since the 1960s [32].

### Limitations

As participant recruitment was conducted in primary care centres, participants were those with a greater predisposition towards accepting modern medical science. Their majority opinion on modern medical science as the best pathway of care for mental health problems may therefore not be representative of the Javanese population. The study was only successful in exploring the perceptions of Javanese people who were already more amenable to modern medical science. Their answers represent the confluence of cultural beliefs, religious beliefs, and modern medicine. Exploring and understanding their perception helps shape future efforts to improve the mental health system. Participants were considered non-specialist audience and therefore not prompted to consider the distinction between psychotic disorders and affective disorders. Participants were therefore at liberty to interpret what the interviewers meant by “mental health problems”.

Where time and budget are not constraints, an ethnographic study could provide the richness of cultural traditions, norms, and ideas which our semi-structured interviews failed to capture. We further acknowledge the limitation of our interviewers in probing participants on Christian religious practices, despite our participants’ proportion of Muslim vs non-Muslims mirroring the country’s distribution of religion. As all three interviewers were Muslims, we feel that adherence to Islamic religious practices was adequately explored, but the same may not be true for non-Muslim interviewees (12%). Examining the demographic information of our participants further, we acknowledge that we lack representation from Buddhist, Hindu, and other religious minorities.

Given that 87.2% of the Indonesian population identify as Muslim, our sample proportionally represents the population, and our findings are still largely generalisable. Future studies should purposefully include minority perspectives and examine if there are religious differences in the perceived aetiology of mental health problems and the perceived best pathway of mental health care. Our findings also indicate that men might more attuned to the potential of economic or environmental factors or biological factors to cause mental health problems. It might be interesting to further explore these gender differences in future studies.

## Conclusions

This study successfully explored the interplay between the need for healthcare on one hand, as well as traditional beliefs, stigma, and the remnants of a colonial health system on the other hand. The themes which transpired from the interviews indicate a wide variety of preconceptions of mental health problems and assumptions regarding the appropriate treatment pathway. Our findings suggest the shifting perception of the appropriate care pathways for mental health problems among the Javanese relative to previous studies conducted in the 1980s [10]. The interviews show that the majority of Javanese people in this study rely significantly less on traditional healers to manage mental health problems than in the past. Our exploration shows that this shift was facilitated by religious leaders and a desire to practise the Islamic faith in its pure form, further highlighting the importance to be socially accepted among the Javanese.

Our results indicate participants’ understanding of the causes of mental health problems. We anticipated this to be different from expert (or learned audience) knowledge on what is mental health, underlining the need for mental health education for the wider public. Even among experts, there remains disagreements and some may argue inconclusive evidence of the causes of mental illness. This study directly contributed to understanding the motivations of patients and how a one-size-fits-all approach, such as the nation-wide service expansion following the WHO mhGAP framework, should be appropriately contextualised to fit with the often-shifting cultural norms of its target audience.

In line with the recent shift of focus within global mental health towards the lived experiences of illness where clinicians are expected to understand the illness as the patient understands, feels, perceives, and responds to it [23], our findings are relevant to future efforts of mental health promotion and government policy on mental health service provision. We underline the importance of appropriate training for frontline healthcare professionals on the necessity of meeting the spiritual needs of patients in the clinical setting, especially considering a growing body of evidence that spirituality is beneficial to mental health and recovery. Understanding the socio-cultural context of mental health problems, which may vary considerably between genders and age groups, has become a necessity in the effort to improve the accessibility of mental health services and treatment adherence, therefore bridging the mental health treatment gap.

## Supplementary Information

Additional file 1. Interview Topic Guide (as approved by steering committee at The University of Cambridge) which was translated to Bahasa Indonesia before participant recruitment.

## Data Availability

Data will be made available upon request to the lead author.
