# Supplementary material for: Perceived causes of mental illness and views on appropriate care pathways among Indonesians
Source: Int J Ment Health Syst. 2021 Sep 23;15:74. doi: 10.1186/s13033-021-00497-5 (PMC8461980; doi:10.1186/s13033-021-00497-5)
Supplement: Supplementary file 1 — Additional file 1. Interview Topic Guide (as approved by steering committee at The University of Cambridge) which was translated to Bahasa Indonesia before participant recruitment. [file 13033_2021_497_MOESM1_ESM.docx]

SEMI-STRUCTURED INTERVIEW TOPIC GUIDE

**SOCIO-CULTURAL FACTORS BEHIND UNDERSTANDING OF MENTAL HEALTH CARE**

**Patient flow during the data collection.**

1. All adult patients pick up a queue number and GHQ-12 + socio-demographic questionnaire from the registration counter.
2. Patients complete the GHQ-12 + socio-demographic questionnaire while waiting for blood pressure measurement.
3. Research Assistants score the GHQ-12 and identify patients who meet the screening criteria.
4. Patients who meet the screening criteria will be invited to participate in the study. Here patients have the opportunity to say yes or no.

If patients say **yes**:

5. Patients will be given Information Sheet, Consent Form, CSRI, WHODAS 2.0, and EQ5D.

6. Patients will be seen by a GP or Clinical Psychologist for psychiatric interview.

7. Patients will be waiting for their prescription or payment (mental health care is free). While waiting, they will be interviewed for their "socio-cultural determinants of decision making" using the topic guide below.

If patients say **no**:

5. Patients will be seen by a GP for their other ailments or concerns.

6. Patients will be waiting for their prescription or payment. While waiting, they will be invited to participate in the qualitative study.

7. Patients who agree to participate will be given Information Sheet, Consent Form, and the interviewed for their "socio-cultural determinants of decision making" using the topic guide below.

**LET THE INTERVIEWEE TELL THEIR STORY AND USE THE QUESTIONS BELOW AS PROBES/REMINDERS**

(Interviewees are patients who met the GHQ-12 screening criteria)

(Part 1) ESTABLISH IF INTERVIEWEE RECEIVED OR REJECTED MENTAL HEALTH CARE AND WHY

**When you were waiting for your queue number earlier, you filled up a short survey. The Research Assistant tried to explain your results, and asked if you want to receive free mental health care.**

(the interviewer will need to show a sample GHQ-12)

**What do you think about your survey result?**

(clarify if patients accepted the offer psychiatric interviews by GPs and/or Psychologists as well as treatment)

(Part 2) CONSIDERATIONS BEHIND DECISION

**What considerations did you have prior to accepting/rejecting the offer for mental health care?**

**Probe:** Let the interviewee tell you their personal reasons. Additionally, ask them to consider:

- Impact of illness on their person, immediate family, workplace/other settings
- Finances (free treatment, afraid can’t afford treatment)
- Quality of care offered
- Awareness of psychological wellbeing

Only if mentioned:

- Stigma (self-held, immediate family, others)
- Any other factors which may arise during the interview

**If you or a family member had any concerns about wellbeing and mental health, where would you go first for help/advice?**

**Probe:** Ask the interviewee to tell you what kind of concerns they have had in the past.

**Prompt:** Why they would go where they have stated?

**Have you had any experiences with traditional healers?**

**Probe:** Ask the interviewee to describe situations or events which led him/her to go to a traditional healer, or any of their family members.

**Prompt:** If you or your family members had seen a traditional healer, did your/their situation improve? Was the effect long lasting?

(Part 3) INTERVIEWEE’S UNDERSTANDING OF MENTAL HEALTH

**Given your screening result earlier, do you think there is a situation to be worried about?**

**What does your religion say about your current situation?**

**What is mental health, to you?**

(Part 4) **FINALLY:**

**Do you have anything else that you would like to raise that we have not covered in this questionnaire?**

**Do you have any further questions that you would like to ask me?**

**Thank you for your time.**
